# Amine oxidase copper-containing 3 (AOC3) inhibition: a potential novel target for the management of diabetic retinopathy

**DOI:** 10.1186/s40942-021-00288-7

**Published:** 2021-04-12

**Authors:** David S. Boyer, Joerg F. Rippmann, Michael S. Ehrlich, Remko A. Bakker, Victor Chong, Quan Dong Nguyen

**Affiliations:** 1 Retina-Vitreous Associates Medical Group, Los Angeles, CA USA; 2 CardioMetabolic Diseases Research, Boehringer Ingelheim Pharma, Biberach an der Riss, Germany; 3 Boehringer Ingelheim Pharmaceuticals Inc., Ridgefield, CT USA; 4 Boehringer Ingelheim International GmbH, Ingelheim Am Rhein, Germany; 5 Byers Eye Institute, Stanford University, Palo Alto, CA USA

**Keywords:** Amine oxidase copper-containing 3, Semicarbazide-sensitive amine oxidase, Vascular adhesion protein 1, Non-proliferative diabetic retinopathy

## Abstract

**Background:**

Diabetic retinopathy (DR), a microvascular complication of diabetes, is the leading cause of visual impairment in people aged 20–65 years and can go undetected until vision is irreversibly lost. There is a need for treatments for non-proliferative diabetic retinopathy (NPDR) which, in comparison with current intravitreal (IVT) injections, offer an improved risk–benefit ratio and are suitable for the treatment of early stages of disease, during which there is no major visual impairment. Efficacious systemic therapy for NPDR, including oral treatment, would be an important and convenient therapeutic approach for patients and physicians and would reduce treatment burden. In this article, we review the rationale for the investigation of amine oxidase copper-containing 3 (AOC3), also known as semicarbazide-sensitive amine oxidase and vascular adhesion protein 1 (VAP1), as a novel target for the early treatment of moderate to severe NPDR. AOC3 is a membrane-bound adhesion protein that facilitates the binding of leukocytes to the retinal endothelium. Adherent leukocytes reduce blood flow and in turn rupture blood vessels, leading to ischemia and edema. AOC3 inhibition reduces leukocyte recruitment and is predicted to decrease the production of reactive oxygen species, thereby correcting the underlying hypoxia, ischemia, and edema seen in DR, as well as improving vascular function.

**Conclusion:**

There is substantial unmet need for convenient, non-invasive treatments targeting moderately severe and severe NPDR to reduce progression and preserve vision. The existing pharmacotherapies (IVT corticosteroids and IVT anti-vascular endothelial growth factor-A) target inflammation and angiogenesis, respectively. Unlike these treatments, AOC3 inhibition is predicted to address the underlying hypoxia and ischemia seen in DR. AOC3 inhibitors represent a promising therapeutic strategy for treating patients with DR and could offer greater choice and reduce treatment burden, with the potential to improve patient compliance.

## Background

Diabetic retinopathy (DR) is a highly specific neurovascular complication affecting patients with type 1 and type 2 diabetes mellitus (DM) [1]. DR frequently affects both eyes, but may be asymptomatic during its early stages [1, 2]. As the disease progresses, an individual’s vision may become blurred and their visual field restricted [2]. Ultimately, DR can progress to sight-threatening stages, the leading cause of new blindness among people of working age in developed countries [1]. Both tissue injury and vascular events contribute to vision loss in DR [1, 3], and neurodegeneration due to diabetes itself is an early event in the pathogenesis of DR [4].

Increased vascular permeability and/or capillary non-perfusion in DR leads to diabetic macular edema (DME) and macular ischemia, impairing the individual’s central vision [3, 5]. However, while the pathophysiology of DME and DR are highly interconnected, they may also develop independently [6]. Pre-retinal or vitreous hemorrhage stemming from angiogenesis is associated with damage to retinal neurons in the proliferative phase [3]. Moreover, vitreous contraction creates retinal distortion and tractional detachments, which can be severe and irreversible [1, 3].

Although the optimization of glycemic control, blood pressure, and serum lipid control is recommended to slow the progression of DR and decrease the risk of vision loss, many patients still develop proliferative diabetic retinopathy (PDR) [1]. While blindness in DR is largely preventable and moderate ocular abnormalities may even be reversible with systemic control (HbA1c) [7], treatment for DR is not usually administered until vision loss becomes apparent. Unfortunately, in the USA, only around 35–60% of patients with DM receive adequate DR screening [8, 9], and only 30–49% obtain appropriate follow-up care [10]. International guidelines advise that non-proliferative diabetic retinopathy (NPDR) should not be treated unless clinically significant macular edema is present [11]. PDR is characterized by pathologic vitreoretinal neovascularization, while NPDR comprises hallmark features of intraretinal hemorrhages, microaneurysms, lipid exudates, and capillary nonperfusion [12, 13]. DR severity is frequently graded in clinical studies using the Diabetic Retinopathy Severity Scale (DRSS); DRSS is derived from the ETDRS (Early Treatment Diabetic Retinopathy Study) and classifies mild, moderate, moderately severe, and severe NPDR as levels 35, 43, 47, and 53, respectively [14, 15] (Table 1).

**Table 1:** Correlation between disease severity levels according to the DRSS and ETDRS classification of DR

Disease severity [16]	Findings observable upon dilated ophthalmoscopy [16]	ETDRS level equivalent [16]	Risk assessment [16]	Prevalence of vision-related difficulty [17]
No apparent retinopathy	No abnormalities	Level 10: DR absent		Functional burden: 20.2% Reading: 11.7% Noticing objects to the side: 4.4%
Mild NPDR	Microaneurysms only	Level 20: Very mild NPDR		Functional burden: 20.4% Reading: 8.5% Noticing objects to the side: 2.8%
Moderate NPDR	More than just microaneurysms but less than severe NPDR	Levels 35, 43: Mild, moderate NPDR less than 4:2:1	One-year risk for early PDR: 5.4–11.9% One-year high-risk PDR: 1.2–3.6%	
		Level 47: Moderately severe NPDR less than 4:2:1	One-year risk for early PDR: 26.3% One-year high-risk PDR: 8.1%	
Severe NPDR	No signs of PR but with any of the following: Extensive (> 20) intraretinal hemorrhages in each of the 4 quadrants Definite venous beading in ≥ 2 quadrants Prominent IRMA in ≥ 1 quadrant	Levels 53A–53E: Severe to very severe NPDR, 4:2:1 rule	One-year risk for early PDR: 50.2% (severe NPDR) One-year high-risk PDR: 14.6% (severe NPDR)–45.0% (very severe NPDR)	Functional burden: 48.5% Reading: 33.3% Noticing objects to the side: 14.5%
PDR	≥ 1 of the following: Neovascularization Vitreous/preretinal hemorrhage	Levels 61, 65, 71, 75, 81, 85: PDR, high-risk PDR, very severe, or advanced PDR		
Ungradable		Level 90: Cannot grade, even sufficiently for level 81 or 85		

*DRSS* Diabetic Retinopathy Severity Scale, *DR* diabetic retinopathy, *ETDRS* Eary Treatment Diabetic Retinopathy Study,* IRMA* intraretinal microvascular abnormalities, *NPDR* non-proliferative diabetic retinopathy, *PDR* proliferative diabetic retinopathy

## Current treatment options for moderately severe and severe NPDR

While most patients with moderately severe or severe NPDR (DRSS level 47–53) do not receive treatment, these are not benign conditions and therapeutic intervention before the development of PDR, vitreous hemorrhage, or DME could reduce visual loss. The early detection of moderately severe to severe NPDR is especially important as the risk of vision loss increases with disease progression. The risk of developing early PDR after 1 year is 26.3% and 50.2% in patients with DRSS levels of 47 and 53, respectively [16]. Thus, regular screening can help to identify those patients at high risk of rapid progression who would benefit from treatment [1]. In the Phase 3 PANORAMA trial, 53% of patients with severe NPDR and 36% with moderately severe NPDR in the untreated control group developed vision-threatening events, compared with 15% of patients with severe NPDR and 8–10% of patients with moderately severe NPDR in the groups given intravitreal (IVT) treatment with the vascular endothelial growth factor A (VEGF-A) inhibitor aflibercept [18]. Treatment delays in patients who received a sham control before crossing over to ranibizumab in the extension phase of the RISE and RIDE studies also gained fewer ETDRS letters in best-corrected visual acuity score at Month 36 compared with baseline versus those who received ranibizumab throughout the studies [19].

Direct ophthalmic treatment options for NPDR are limited to pan-retinal photocoagulation (PRP), IVT corticosteroids, and IVT VEGF inhibitors. A Cochrane database systematic review of four trials of PRP and one trial of selective photocoagulation of non‐perfusion areas concluded that laser photocoagulation is beneficial for PDR, but the authors noted the paucity of high-quality evidence confirming this conclusion [20]. However, the recent Diabetic Retinopathy Clinical Research Network (DRCR.net) Protocol S study showed that more patients with PDR treated with PRP had vitreous hemorrhages compared with patients receiving ranibizumab [21]. There is some evidence to show that PRP may reduce visual acuity in severe NPDR [22], and a systematic review and economic evaluation found insufficient evidence to recommend PRP in this patient group [23]. PRP sessions are uncomfortable and macular edema may develop or worsen after treatment [23, 24]. In addition, patients who undergo PRP treatment may experience a constricted visual field, reduced night vision, and macular or lens damage [24].

IVT implants of corticosteroids are licensed for the treatment of DME, but their use in DR remains limited as it may be associated with an increase in intraocular pressure and cataract development or worsening [25–29].

The VEGF inhibitors ranibizumab and aflibercept are approved by the US Food and Drug Administration (FDA) for the treatment of DR [30], and bevacizumab is widely used off-label for DME [25]. Brolucizumab, which is FDA approved for neovascular age-related macular degeneration [31], is also under investigation in PDR and DME [32, 33]. IVT VEGF inhibitor injections are invasive, expensive, and can be inconvenient to patients and their caregivers; at times, it may be necessary to treat each eye at a separate visit, thereby doubling treatment, travel, and recovery times, all of which contribute to patient, caregiver, and healthcare system burden [34, 35]. In addition, VEGF inhibitors do not improve ischemia or inflammation, do not provide neuroprotection, and continued VEGF inhibitor treatment is required to sustain any benefit to ischemia-related diabetic retinal dysfunction [35–38]. Furthermore, not all patients respond meaningfully to VEGF inhibitors [39–41]. In a study of DME among patients with NPDR, approximately three-quarters of eyes did not fully respond to aflibercept (108/141), bevacizumab (106/131), or ranibizumab (111/151) at 2 years [41]. If appointments for VEGF inhibitor administration are missed (non-compliance), e.g. due to illness, DR could progress to more severe disease and may result in irreversible sight loss [37].

There is evidence that treatment with an additional drug with a different mode of action may reduce the effects of DR [42]. There are several barriers to compliance to IVT DR therapy and follow up, including lack of DR knowledge, DM end-organ damage, economic burden (both DR and DM), long appointment waiting times, and inability to attend appointments due to work or illness [43–45]. Given that compliance to IVT VEGF inhibitor therapy is suboptimal even in patients with age-related macular degeneration and those with DME [46], compliance is likely to be even worse in less symptomatic patients with NPDR.

There is substantial unmet need for treatments that target moderately severe and severe NPDR before sight is compromised. If DR progresses to center-involving diabetic macular edema (CI-DME), patients will need long-term, invasive treatment to prevent further disease progression, which may not restore visual acuity [47]. The DRCR.net Protocol S study of 305 patients with PDR treated with PRP or VEGF inhibitor demonstrated that, at 6 months, the VEGF inhibitor ranibizumab failed to prevent neovascularization in 45% of eyes; however, a reduction in hemorrhage and vitrectomy were also identified in the anti-VEGF-treated patients compared with the PRP-treated patients [21]. The related DRCR.net Protocol V study showed that at 2 years, among 702 patients with CI-DME and good visual acuity (20/25 or better), there was no difference in visual acuity loss among those who received aflibercept, laser photocoagulation, or observation (no intervention) [48].

Encouraging patients to become more active in managing their disease is increasingly important, particularly in DM [49]. Although oral treatments for DR may be more convenient for patients than an IVT injection, they are not without possible drawbacks as patients may forget to take oral agents. In addition, compliance can be tracked more readily by physicians with an IVT injection, as the injection is administered in a clinic compared with self-administration of oral medication at home. Furthermore, with an oral agent for NPDR, the potential for systemic effects, both good and bad, must be considered. Nevertheless, efficacious systemic therapy for NPDR, including oral treatment, may provide a convenient therapeutic approach for patients and physicians, and would reduce the treatment burden for patients and their caregivers.

## The role of inflammation in DR

The neurovascular unit describes the interplay between blood vessel endothelial cells and pericytes, astrocytes, Müller cells, and neurons to establish the blood–retinal barrier (BRB), which controls nutrient flow to–and the removal of metabolic waste products from–the neural retina [3]. Müller glial cells are a significant source of inflammatory modulators, suggesting that retinal glial cell activation may play an early role in the onset of inflammatory processes that damage the retina as the disease progresses [50]. Elevated levels of inflammatory cytokines such as interleukin (IL)-1β, IL-2, IL-4, IL-5, IL-6, IL-8, interferon γ (IFNγ), and tumor necrosis factor α (TNFα) are found in the sera or vitreous or aqueous humor of patients with DR [51, 52]. Levels of IL-8 and TNFα are even higher among patients with NPDR compared with PDR [51]. Progressive retinal injury may reduce the integrity of the BRB leading to an accumulation of inflammatory and angiogenic mediators within the vitreous cavity [3]. The build-up of IL-1β and TNFα induces the expression of intracellular adhesion molecule 1 (ICAM1) and vascular cell adhesion molecule 1 (VCAM1), which attract leukocytes leading to leukostasis [53]. A further consequence of BRB impairment is the migration of macrophages into the neurosensory retina and/or an increased adherence to the vasculature [3].

## The potential role of corticosteroids in DR

The importance of inflammation in the pathogenesis of DR provides a rationale for using corticosteroids as a treatment strategy–targeting the synthesis of key proinflammatory mediators such as IL-6, IL-8, TNFα, ICAM1, and VEGF [54]. Several corticosteroids have been extensively studied in the treatment of DME, including triamcinolone acetonide (TA), the fluocinolone acetonide (FA) implant, and the dexamethasone (DEX) implant [26–29, 55, 56]. However, their clinical impact has been less thoroughly assessed in DR.

In an exploratory analysis of data from a DRCR.net Phase 3 trial in DME, IVT TA appeared to reduce the risk of DR progression compared with focal/grid photocoagulation alone [57]. A subsequent exploratory analysis in eyes without PDR at baseline suggested that the 3-year cumulative probability of retinopathy worsening may be higher in TA-treated eyes compared with those treated with the VEGF inhibitor ranibizumab [58]. However, in patients with PDR both treatments were associated with a reduced risk of PDR worsening compared with sham therapy [58]. More recently, a post hoc analysis of data from two Phase 3 DME trials suggested that another corticosteroid treatment, the FA implant, can delay the development of PDR and slow DR progression compared with sham control [59]. Finally, a retrospective cohort study of 60 patients with NPDR and DME found that treatment with the DEX implant could delay DR progression and improve DR severity over 24 months [60].

However, although these data are promising, prospective, randomized trials are lacking. In particular, studies comparing corticosteroid treatment with a VEGF inhibitor in patients with NPDR would be valuable. Furthermore, it is not known whether the current data on patients with DR and DME are applicable to those without DME. Known adverse events of corticosteroids, such as increased interocular pressure, glaucoma, and cataracts [28, 57, 60], could be limiting their widespread use and restricting their use in patients with severe DR and/or those who are pseudophakic.

## The role of AOC3 protein in retinal inflammation and neovascularization

**Fig. 1:**
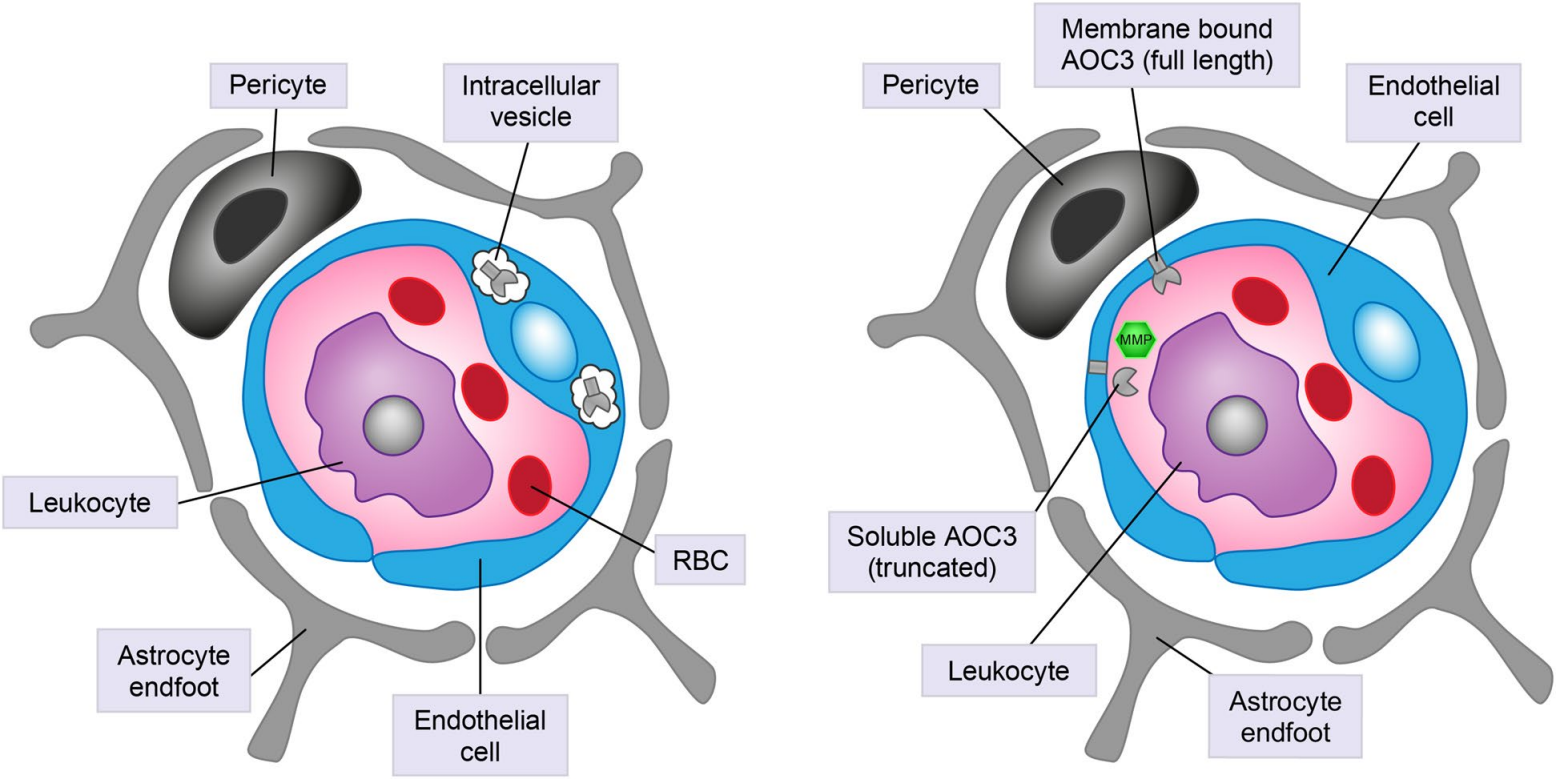
Amine oxidase copper-containing 3 (AOC3) is stored in intracellular vesicles and rapidly translocates to the luminal surface of endothelial cells during inflammation. Matrix metalloproteinases (MMPs) release a proportion of membrane-bound AOC3 resulting in a soluble, truncated form. Both membrane-bound and soluble AOC3 are enzymatically active. *RBC* red blood cell

**Fig. 2:**
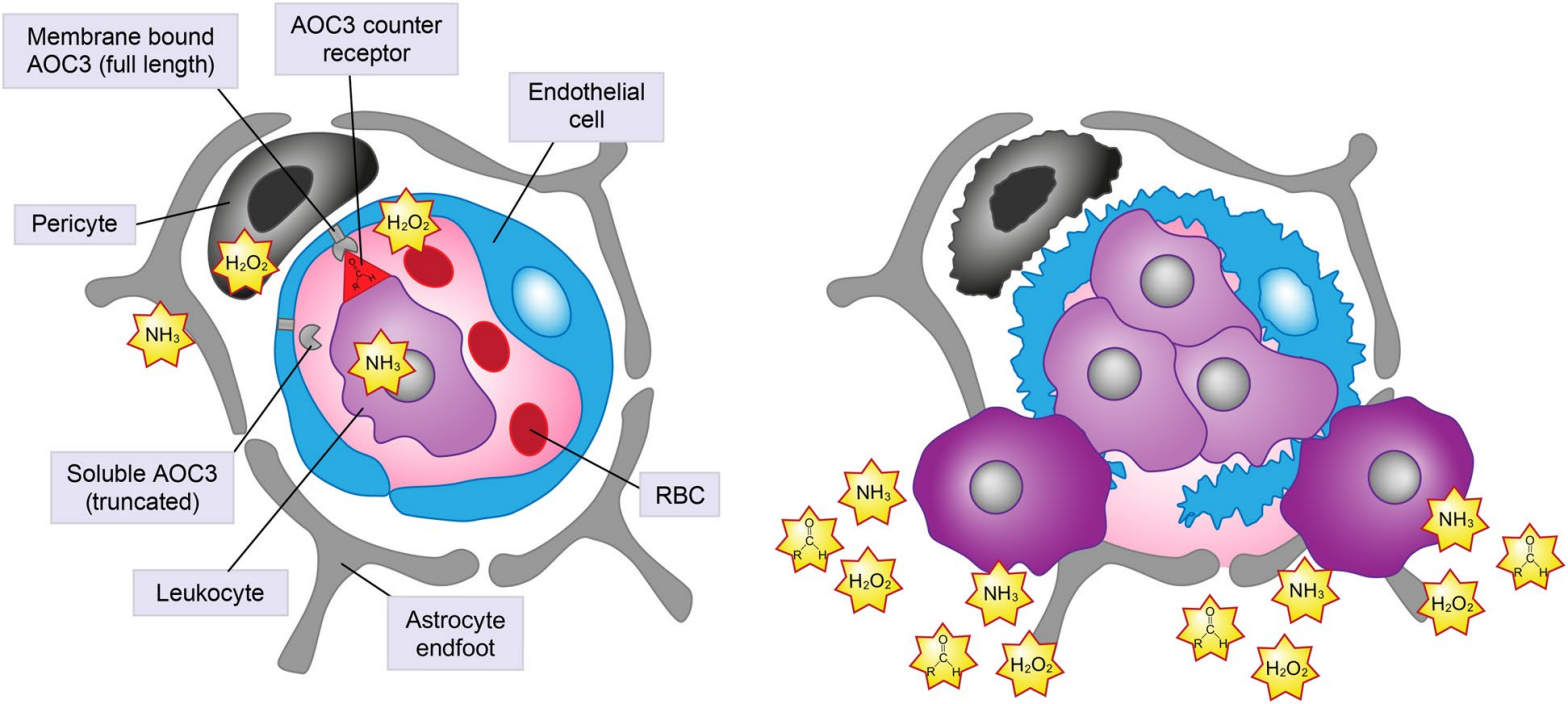
When amine oxidase copper-containing 3 (AOC3) on the retinal endothelium binds to the AOC3 counter receptor on the leukocyte, aldehydes, ammonia, and reactive oxygen species are released, which contribute to inflammation. Leukostasis is triggered and leukocytes rupture the blood vessel leading to ischemia. Soluble AOC3 does not mediate leukostasis but contributes to the release of ammonia and reactive oxygen species. *RBC* red blood cell

Amine oxidase copper-containing 3 (AOC3), also known as semicarbazide-sensitive amine oxidase and vascular adhesion protein 1 (VAP1), is a membrane-bound adhesion protein that facilitates the binding of leukocytes to endothelial cells and their subsequent transmigration to sites of inflammation (Figs. 1 and 2) [61]. AOC3 is abundant in the activated endothelium of vascularized tissues, including the retina, kidney, and liver [62, 63], and is associated with vascular diseases and inflammatory conditions such as chronic kidney disease, chronic liver disease, and DM [61, 64–69]. AOC3 has previously been targeted for therapeutic intervention in autoimmune and inflammatory diseases, and several inhibitory compounds have been investigated over the past two decades [70–73]. Furthermore, AOC3 has been implicated as a possible candidate protein for mediating inflammation and oxidative stress in PDR [74], and may be important in the activation of early events in NPDR [68, 74, 75]. AOC3 is stored in intracellular vesicles and rapidly translocates to the luminal surface of endothelial cells during inflammation [76]. A soluble form of AOC3, which retains enzymatic activity, may also be released by shedding from the endothelial cell membrane following cleavage with matrix metalloproteinases (MMPs) (Fig. 1) [74]. AOC3 on the retinal endothelial cells mediates slow rolling, firm adhesion, and transmigration of leukocytes, triggering leukostasis [61, 77]. Aldehydes, ammonia, and reactive oxygen species are released as a result of the interaction between AOC3 and its substrates, which contribute to inflammation and the accumulation of leukocytes, which rupture the blood vessel leading to ischemia [76]. Although soluble AOC3 does not mediate leukostasis, it contributes to the release of ammonia and reactive oxygen species (Fig. 2). Elevated levels of soluble AOC3 are found in type 1 and type 2 DM, correlating with complications such as end-stage renal disease, cardiovascular mortality, and DR [76, 78]. Furthermore, high levels of soluble AOC3 are predictive of cancer risk in patients with type 2 DM [79].

**Fig. 3:**
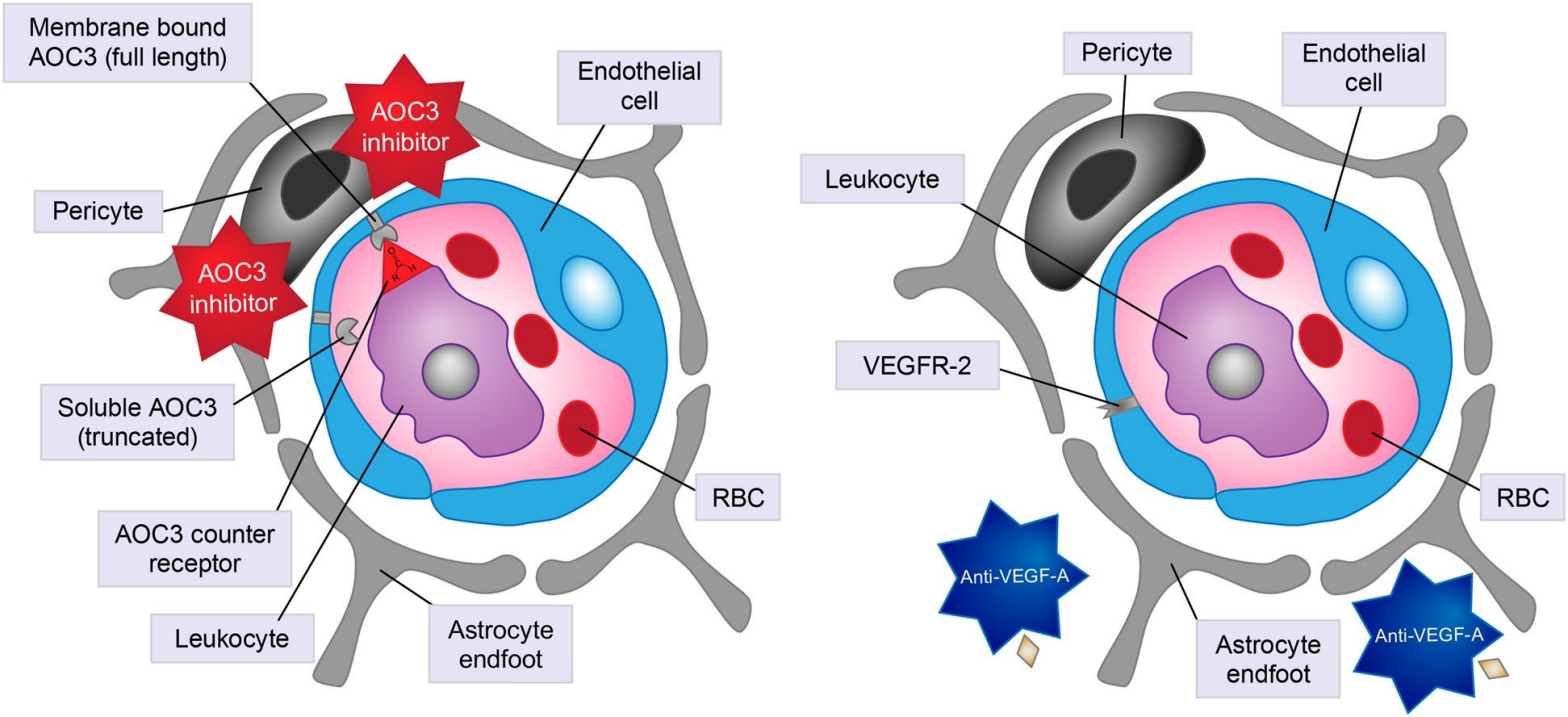
Amine oxidase copper-containing 3 (AOC3) inhibition of membrane-bound form reduces leukocyte recruitment and thereby transmigration. A further consequence of reduced AOC3 enzymatic activity via inhibition of the membrane-bound and soluble forms is likely to be a decrease in the oxidative deamination of primary amines, thus reducing the production of aldehydes, ammonia, and hydrogen peroxide, and ultimately reducing reactive oxygen species. Vascular endothelial growth factor (VEGF) inhibitors bind to the receptor-binding site on VEGF-A, which inhibits interaction with, and activation of, VEGF receptor 2 (VEGFR-2) on the surface of endothelial cells, thereby reducing angiogenesis. *RBC* red blood cell

Inhibition and blocking of membrane-bound AOC3 were shown in preclinical models to reduce leukocyte recruitment and thereby transmigration [61, 80, 81]. A further consequence of reduced AOC3 enzymatic activity (of both full length and soluble forms) is a decrease in the oxidative deamination of primary amines, thus reducing the production of aldehydes, ammonia, and hydrogen peroxide [82], and ultimately reactive oxygen species (Fig. 3). Because the neurovascular unit of the retina is highly susceptible to hyperglycemic stress, inflammation, and reactive oxygen species, AOC3 inhibition may improve vascular function and lead to improved DRSS grading. Furthermore, leukocytes secrete proinflammatory cytokines, and therefore AOC3 blockade is predicted to reduce cytokine expression, macrophage recruitment, and choroidal neovascularization [81].

**Fig. 4:**
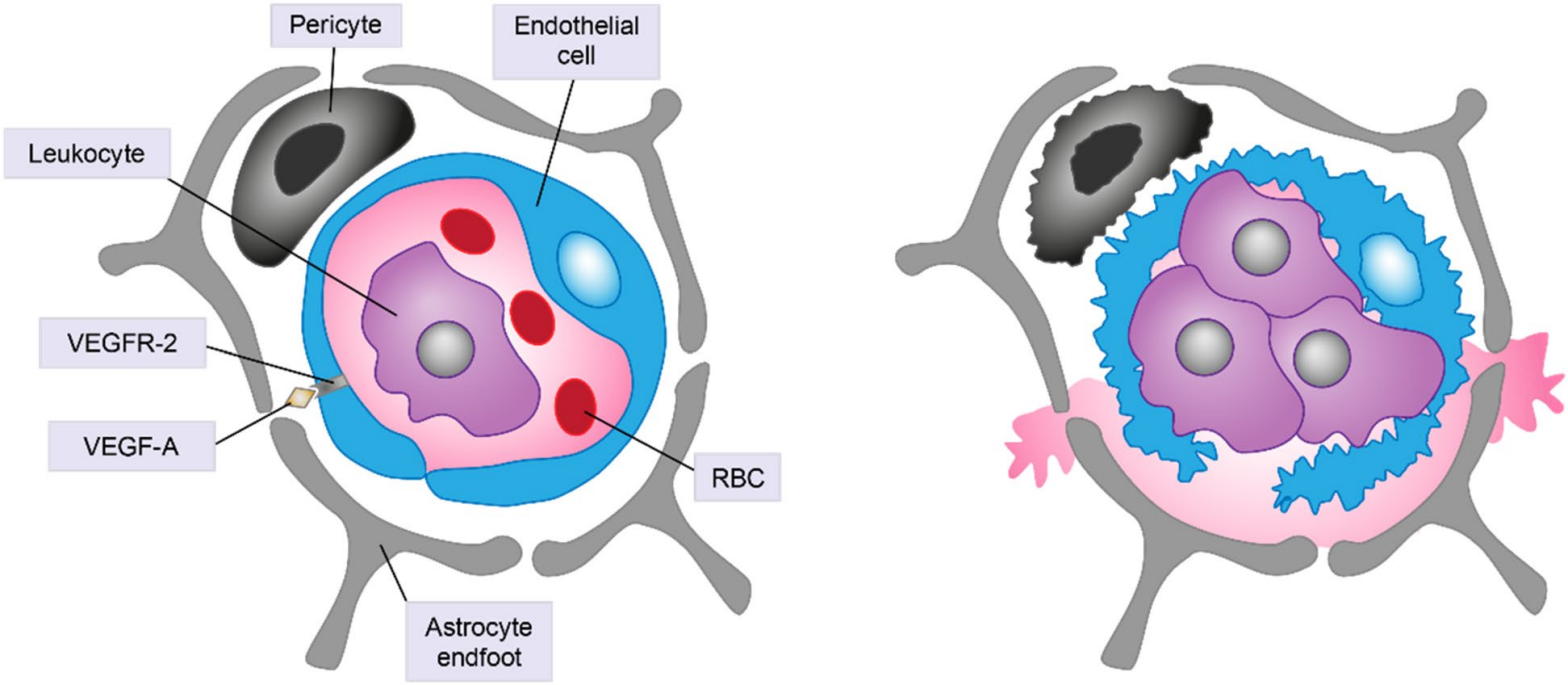
Capillary obstruction due to leukostasis and capillary dropout due to death of vascular pericytes and endothelial cells lead to tissue ischemia. Resulting hypoxia may drive expression of vascular endothelial growth factor-A (VEGF-A). VEGF-A promotes vascular permeability resulting in leakage of plasma through the blood–retinal barrier (BRB) and tissue edema through its interactions with VEGF receptor 2 (VEGFR-2) on vascular endothelial cells. *RBC* red blood cell

In addition, tissue ischemia and the resulting hypoxia in DR drive expression of VEGF-A [83]. VEGF-A promotes vascular permeability resulting in leakage of plasma through the blood-retinal barrier (BRB) and tissue edema through its interactions with VEGF receptor 2 (VEGFR-2) on vascular endothelial cells (Fig. 4) [83]. All currently available VEGF inhibitors bind to the receptor-binding site on VEGF-A, which inhibits interaction with, and activation of, VEGFR-2 on the surface of endothelial cells and, in turn, reduces angiogenesis, ultimately preventing progression and improving DRSS/ETDRS grading [84, 85]. Aflibercept and conbercept also act to scavenge placental growth factor (PIGF) [85, 86], which may lead to an increase in efficacy because PIGF can indirectly stimulate angiogenesis by binding to VEGFR-1, thus increasing the availability of VEGF-A to activate VEGFR-2 [84]. However, VEGF inhibitors do not reduce inflammation or reactive oxygen species (Fig. 3). Instead, they act on the consequence of the disease–elevated secretion of VEGF–without addressing the actual cause of the disease. Consequently, such treatments are unlikely to result in sustained improvements in DR as they are not expected to ameliorate the underlying hypoxia or ischemia.

## Therapeutic targeting of AOC3 in DR

To date, three orally administered AOC3 inhibitors have been investigated in DR and related conditions: ASP8232, RTU-1096, and BI 1467335 (formerly PXS-4728A) (Table 2) [87–91]. Given that DR shares aspects of its pathogenesis with related conditions, such as DME, success in these related indications may support potential efficacy in DR.

**Table 2:** Summary of AOC3 inhibitor preclinical and clinical findings in DR and DME

Agent	Preclinical findings	Clinical findings in DR	Other therapeutic areas of interest
ASP8232	Inhibited plasma VAP1 (AOC3) activity and improved retinal hyperpermeability and plasma total antioxidant status in rat DME model. More effective in reducing ocular hyperpermeability when combined with IVT anti-rat VEGF antibody than either agent alone [87]	VIDI study Phase 2a–CI-DME. Failed to reduce CST alone, no additional benefit in combination with ranibizumab [87]	Reduced residual albuminuria, a surrogate marker for disease progression, in patients with type 2 DM and CKD [88]
RTU-1096	Abrogated outer nuclear layer thickening and reduced upregulation of ICAM1 in mice after retinal laser photocoagulation [91]	Phase 1–healthy volunteers. Well tolerated at all tested doses [93]	
BI 1467335 (formerly PXS-4728A)	Inhibited neutrophil tethering and rolling, reduced inflammation in mouse models [89, 90]	ROBIN (NCT03238963) Phase 2a–NPDR. Primary safety endpoint met, unable to demonstrate a clear efficacy signal (development discontinued due to risk of DDI) [94, 95]	NCT03166735 Phase 2a–NASH. Results pending (development discontinued due to risk of DDI) [97, 98]

*CI-DME* center-involving diabetic macular edema, *CKD* chronic kidney disease, *CST* central subfield thickness, *DDI* drug–drug interaction, *DM* diabetes mellitus, *DME* diabetic macular edema, *ICAM1* intracellular adhesion molecule 1, *IVT* intravitreal, *NASH* non-alcoholic steatohepatitis, *NPDR* non-proliferative diabetic retinopathy; *ROBIN* Randomized, double-masked, placebo-controlled exploratory study to evaluate safety, tolerability, pharmacodynamics and pharmacokinetics of Orally administered BI 1467335 for 12 weeks with a 12 week follow up period in patients with Non-proliferative diabetic retinopathy without center-involved diabetic macular edema, *VAP1* vascular adhesion protein 1, *VIDI* VAP1 inhibition in diabetic macular edema

Initial findings with ASP8232 were promising: pre-clinical studies demonstrated improved retinal and ocular hyperpermeability and reductions in surrogate markers of disease progression were observed in patients with type 2 DM [87, 88]. However, in the VIDI (VAP1 inhibition in DME) study, ASP8232 failed to reduce central subfield thickness in patients with CI-DME when administered alone, and no additional benefit was observed when combined with ranibizumab [87]. The trial investigators postulated that the presence of significant CI-DME may require inhibition of VEGF for clinical benefit, whereas DR could be reduced by AOC3 inhibition alone; they also suggested that alternative delivery modes may warrant further investigation [87]. It should be noted that, based on non-linear mixed effects exposure–response pharmacodynamic modeling, the effects of ASP8232 are reversible, and acute effects at clinical doses in other indications (i.e. diabetic kidney disease) may have been artefactual [92].

In a mouse model of retinal inflammation, systemic administration of RTU-1096 reduced upregulation of ICAM1, an adhesion molecule for leukocytes, in the retina [91]. In Phase 1 trials that completed in 2015, once-daily oral RTU-1096 was well tolerated at all tested doses and produced a sustained reduction in serum AOC3 in healthy male volunteers [93]. However, no trials of RTU-1096 in DR or any other indication are currently registered on ClinicalTrials.gov, and it is unclear why further development was halted.

BI 1467335 inhibits tethering and rolling of neutrophils and reduces inflammation in animal models [89, 90]. The Phase 2a ‘Randomized, double-masked, placebo-controlled exploratory study to evaluate safety, tolerability, pharmacodynamics, and pharmacokinetics of Orally administered BI 1467335 for 12 weeks with a 12 week follow up period in patients with Non-proliferative diabetic retinopathy without center-involved diabetic macular edema’ (ROBIN) study of BI 1467335 completed in May 2020 [94]. The study met its primary endpoint in ocular safety (proportion of patients with any ocular adverse event up to 24 weeks) and BI 1467335 was well tolerated [95]. However, the study was unable to demonstrate a clear efficacy signal (proportion of patients with a  ≥ 2-step improvement in DRSS [from baseline to Week 12]) [95]; this may have been because the trial’s duration (3 months) was too short to demonstrate a clear efficacy benefit. In addition, taking into account recent findings that suggest venous beading (VB) does not respond to treatment with anti-VEGF therapy in patients with NPDR [96], and that VB may differentiate ETDRS level 53 (severe NPDR) from ETDRS level 47 (moderately severe NPDR) in some patients [15], excluding patients with VB may have improved the efficacy signal in the ROBIN study. BI 1467335 was also being investigated in non-alcoholic steatohepatitis (NASH) [97]; the Phase 2a study met pre-specified targets for inhibiting plasma AOC3 activity compared with placebo and clinically relevant changes in NASH biomarkers [98]. However, based on the risk of drug–drug interactions with the compound in patients with NPDR and NASH, which were identified in another Phase 1 study [99], Boehringer Ingelheim announced that the development of BI 1467335 has been discontinued in both indications [95, 98].

## Conclusion

There is substantial unmet need for convenient, non-invasive treatments targeting moderately severe and severe NPDR. By treating the underlying cause of DR rather than its manifestations, resulting improvements may be more sustained. Unlike existing therapies that only target inflammation or angiogenesis, AOC3 inhibition is predicted to reduce leukostasis and reactive oxygen species, ultimately addressing the underlying hypoxia and ischemia seen in DR. Despite recent setbacks in clinical trials, AOC3 inhibition remains an attractive target due to the strength of preclinical data supporting the mechanism of action. The levels of AOC3 inhibition in these trials are likely to have varied among the different agents, and alternative AOC3 inhibitors may be more successful in the future. An orally administered treatment based on either an established or novel mechanism may still be a promising therapeutic strategy for treating patients with DR, with the aim of reducing treatment burden and improving patient compliance.

## Data Availability

Not applicable.

## Abbreviations

AOC3: Amine oxidase copper-containing 3
BRB: Blood–retinal barrier
CI-DME: Center-involving diabetic macular edema
CKD: Chronic kidney disease
CST: Central subfield thickness
DDI: Drug–drug interaction
DEX: Dexamethasone
DM: Diabetes mellitus
DME: Diabetic macular edema
DR: Diabetic retinopathy
DRSS: Diabetic Retinopathy Severity Scale
ETDRS: Early Treatment Diabetic Retinopathy Study
FA: Fluocinolone acetonide
FDA: Food and Drug Administration
ICAM1: Intracellular adhesion molecule 1
IFN: Interferon
IL: Interleukin
IRMA: Intraretinal microvascular abnormalities
IVT: Intravitreal
MMP: Matrix metalloproteinase
NASH: Non-alcoholic steatohepatitis
NPDR: Non-proliferative diabetic retinopathy
PDR: Proliferative diabetic retinopathy
PIGF: Placental growth factor
PRP: Pan-retinal photocoagulation
RBC: Red blood cell
TA: Triamcinolone acetonide
TNF: Tumor necrosis factor
VAP1: Vascular adhesion protein 1
VB: Venous beading
VCAM1: Vascular cell adhesion molecule 1
VEGF: Vascular endothelial growth factor
VEGFR: Vascular endothelial growth factor receptor
